# Serum proteomics of active tuberculosis patients and contacts reveals unique processes activated during *Mycobacterium tuberculosis* infection

**DOI:** 10.1038/s41598-020-60753-5

**Published:** 2020-03-02

**Authors:** Jesús Mateos, Olivia Estévez, África González-Fernández, Luis Anibarro, Ángeles Pallarés, Rajko Reljic, Tufária Mussá, Cremildo Gomes-Maueia, Artur Nguilichane, José M. Gallardo, Isabel Medina, Mónica Carrera

**Affiliations:** 1 Spanish National Research Council (CSIC), Vigo, Pontevedra Spain; 2 Biomedical Research Centre (CINBIO), Galician Singular Center of Research, Galicia Sur Health Research Institute (IIS-GS), Campus Universitario de Vigo S/N, University of Vigo, 36310 Vigo, Pontevedra Spain; 3 Tuberculosis Unit, Infectious Diseases, Internal Medicine Service, Complexo Hospitalario Universitario de Pontevedra, Galicia Sur Health Research Institute (IIS-GS, Pontevedra, Spain; 4 Mycobacterial Infections Study Group (GEIM) of the Spanish Society of Infectious Diseases and Clinical Microbiology (SEIMC), Madrid, Spain; 5 St. George’s, University of London, London, UK; 6 Department of Technologic Platforms, Instituto Nacional de Saúde, Maputo Province, Maputo, Mozambique; 7 Department of Microbiology, Faculty of Medicine, Eduardo Mondlane University, Maputo, Mozambique; 8 Centro de Saúde da Machava II, Maputo Province, Maputo, Mozambique

**Keywords:** Tuberculosis, Biomarkers

## Abstract

Tuberculosis (TB) is the most lethal infection among infectious diseases. The specific aim of this study was to establish panels of serum protein biomarkers representative of active TB patients and their household contacts who were either infected (LTBI) or uninfected (EMI-TB Discovery Cohort, Pontevedra Region, Spain). A TMT (Tamdem mass tags) 10plex-based quantitative proteomics study was performed in quintuplicate containing a total of 15 individual serum samples per group. Peptides were analyzed in an LC-Orbitrap Elite platform, and raw data were processed using Proteome Discoverer 2.1. A total of 418 proteins were quantified. The specific protein signature of active TB patients was characterized by an accumulation of proteins related to complement activation, inflammation and modulation of immune response and also by a decrease of a small subset of proteins, including apolipoprotein A and serotransferrin, indicating the importance of lipid transport and iron assimilation in the progression of the disease. This signature was verified by the targeted measurement of selected candidates in a second cohort (EMI-TB Verification Cohort, Maputo Region, Mozambique) by ELISA and nephelometry techniques. These findings will aid our understanding of the complex metabolic processes associated with TB progression from LTBI to active disease.

## Introduction

Tuberculosis (TB) is currently a global pandemic that kills approximately 1.3 million people worldwide each year^1^. Although the incidence of TB is decreasing each year, the rate of that decline is not high enough to accomplish the TB Global Strategy for the 2015–2035 period^2,3^. *Mycobacterium tuberculosis* (MTB) is the causal agent of the pulmonary TB^4^, which is air-transmitted by nasal/oral inhalation of aerosol droplets carrying the pathogen from an active TB patient to a healthy individual usually through coughing or sneezing^5^. Small droplets that are able to reach the lower lung induce formation of a granuloma^6^, which is a host-defensive structure providing, a fibrotic physical barrier between the infected and the healthy neighboring tissues^7^. Latently infected individuals (LTBI) represent roughly a quarter of the global population^8^. Tuberculin Skin Test (TST) and Interferon-Gamma Release Assay (IGRA) are commonly used for diagnosis^9,10^ of LTBI individuals. Although they cannot infect a healthy person, MTB activation occurs in roughly 10% of cases due to recurrent infections or a compromised immune system in the host^11^.

The safest and most cost-effective way to fight against infectious diseases is vaccination^12^. TB and other poverty-related and neglected infectious diseases still lack effective vaccines to protect the entire population^13^. BCG (Bacillus Calmette-Guerin), the only licensed vaccine currently in use against TB, was developed eighty years ago and displays an efficacy of 80% or more in children under 4 years old^14^. However, the efficacy of BCG in adolescents and adults is variable, 0 to 80% depending on the geographical region^15^. The TB Global Strategy for the period of 2015–2035 draws special attention to the urgent need to develop a new, more efficient vaccine against TB^16,17^. Efforts are being made in different directions. While some research strategies have focused on boosting the existing BCG vaccine^18^ or exploring the use of new BCG strains^19^, others are focused on developing a mucosal vaccine, as is the case with the Horizon2020 EMI-TB consortium^20,21^.

Proteomics has been used in the last decade as a tool to globally analyze, at the protein level, cellular and whole-organism processes related to disease and its progression. Proteomic profiling allows the elucidation of connections between various cellular pathways and thus complements both the genomics and traditional biochemical approaches. Several studies have focused on the MTB proteome, all of which were recently reviewed^22^. Alternatively, for the study of the molecular basis of health and disease, biological fluids from patients and controls are a reliable source for the identification of protein markers^23^. Thanks to the extensive and continuous progress of analytical proteomics and because of the emergence of Mass Spectrometry-based techniques that facilitate the identification quantification and characterization of proteins, proteomics is expected to be, in the near future, the tool of choice for diagnosing patients and searching for therapeutic biomarkers^24,25^.

In the present study our focus was on identifying the specific protein signatures in sera of active TB patients and their contacts, including latently infected (LTBI) and uninfected contacts.

## Experimental Procedures

### Patient selection and database management

Collection (discovery cohort) of samples from TB patients and their contacts (LTBI and uninfected) started in Pontevedra (Spain) on September 2015, within the framework of the EMI-TB project (Eliciting Mucosal Immunity to Tuberculosis) following previously established clinical criteria^26^. Briefly, patients were diagnosed using the highest standards for clinical TB research, including tuberculin skin test (TST) and/or the Quantiferon-TB-Gold test (QFT) and again after 8–12 weeks after the last possible exposure to the index case if the first test was negative. Additionally, chest radiographies were done to exclude active TB patients from the LTBI group. Lowenstein-Jensen and Colletsos culture were done for all TB-diagnosed patients. The study was approved by the Spanish National Bioethics committee (Project ID: 643558). Exclusion criteria were age <18 y, co-infection with the human immunodeficiency virus (HIV), any other immunossuppresive medical condition or concomitant use of immunossuppresive drugs. Patients with previous TB or LTBI were also excluded for the study. Serum samples were collected right after final diagnosys and before commencement of the anti-TB drug treatment. The datasets were analysed in blind fashion using codes and keeping the anonymity of the volunteers. Summary of the demographic data considered relevant to the study is shown in Table 1. Informed consent was obtained, and all patients and contacts received a detailed explanation of the project and its objectives, as well as the ensurance of the confidentiality.

**Table 1:** Demographic summary of the patients included in the EMI-TB shotgun study cohort (Pontevedra, Spain).

Patient Type	Sex	Age (years; mean ±SD)	Contact score (mean ±SD)*	BCG vaccination	MTB culture
Active TB patient (n = 26)	Female (15.4%), male (84.6%)	41.3 ± 13.9	n.a.	Yes: 15.4% (n = 4) No: 84.6% (n = 22)	Positive: 100% (n = 26) Negative: 0% (n = 0)
LTBI contact (n = 29)	Female (41.3%), male (58.7%)	47.4 ± 14.7	10.9 ± 2.6	Yes: 27,6% (n = 8) No: 72.4% (n = 21)	n.a.
Uninfected contact (n = 44)	Female (52.3%), male (47.7%)	40.0 ± 15.2	9.6 ± 2.3	Yes: 32.8% (n = 14) No: 68.2% (n = 30)	n.a.

*“Contact Score” was assigned to the contacts following this criteria: acid-fast bacilli (AFB) microscopy index: from 0 to 4; cavitary X-rays: No = 0, Yes = 1; contact: No = 0, Yes = 1; hours of exposure/day: 0–3 hours: 1, 4–7 hours: 2, 8–11 hours: 3, ≥ 12 hours: 4; type of exposure: Outdoors= 0.25, Different room: 1, As a bar: 2, As a class: 2.5, As an office: 3, As a room or car: 4; weeks in contact with Index Case: <12 weeks: 0, ≥ 12 weeks: 1; sleeps in the same room: No = 0, Yes = 1; first-degree family relationship: No = 0, Yes = 1.

In parallel, and also under the framework of the EMI-TB project, samples from TB patients (verification cohort) and their contacts (LTBI and uninfected) were also collected in the Maputo Region (Mozambique) using the same standard operating procedures (SOPs). The study was approved by the Mozambican National Bioethics committee (IRB:00002657; ID: 298/CNBS/15).

Additionally, all the experimental procedures described in this section were performed in accordance with relevant guidelines and regulations (both national and the EU).

### Sample collection and storage of quantitative shotgun proteomics samples

EDTA (ethylene diamine tetra acetic acid) blood samples (40 mL) from each participant were obtained by the TB unit and immediately transferred to the University of Vigo for processing. During the whole sample collection period the same SOP (Standard Operational Procedure), based on the recommendations of the Human Proteome Organization (HUPO), was applied. Once obtained, the serum samples were distributed into 1000 μL aliquots (Protein LoBind Tube 1.5 mL, Eppendorf, Germany) and stored at −80 °C for later analysis.

### Protein preparation for TMT labeling

Total protein in the serum samples was precipitated by the addition of six volumes of cold acetone and incubated overnight at −20 °C. After centrifugation, the dried protein pellet was resuspended in 0.1 M TEAB buffer solution (Triethyl ammonium bicarbonate: Stock solution: 1 M). The protein concentration was measured by the bicinchoninic acid (BCA) assay. The protein integrity was confirmed in 1 µg aliquots by SDS-PAGE and silver staining. Samples with protein degradation were detected and discarded from the proteomic analysis.

### Experimental design and statistical rationale

For the shotgun study, we analyzed individual serum samples rather than pools and later applied a robust statistical analysis of quantification ratios (Kruskal-Wallis). For this reason, we increased the number of TMT 10plex labels to five (Table 2). Three active TB patients (channels 126, 127 N and 127 C), three infected LTBI contacts (channels 128 N, 128 C and 129 N), three uninfected contacts (channels 129 C, 130 N and 130 C), making a total of 45 individual samples. One standard mix sample were included within each TMT experiment, prepared by mixing equal amounts of proteins for the nine samples included (channel 131).

**Table 2:** Individual serum samples used in the EMI-TB shotgun study.

***TMT 1 Serum***	***SAMPLE***	PO-20	PO-28	PO-38	PO-24	PO-27	PO-36	PO-21	PO-26	PO-44	Standard
	***Group***	TB	TB	TB	LTBI	LTBI	LTBI	Uninfected	Uninfected	Uninfected	Mix
	***TMT-Label***	126	127 N	127 C	128 N	128 C	129 N	129 C	130 N	130 C	131
	***BCG vaccination***	No	No	Yes	No	Yes	Yes	No	No	No	n.a.
***TMT 2 Serum***	***SAMPLE***	PO-16	PO-40	PO-51	PO-30	PO-41	PO-54	PO-34	PO-57	PO-65	Standard
	***Group***	TB	TB	TB	LTBI	LTBI	LTBI	Uninfected	Uninfected	Uninfected	Mix
	***TMT-Label***	126	127 N	127 C	128 N	128 C	129 N	129 C	130 N	130 C	131
	***BCG vaccination***	No	No	No	No	No	No	No	No	No	n.a.
***TMT 3 Serum***	***SAMPLE***	PO-32	PO-63	PO-88	PO-31	PO-42	PO-55	PO-68	PO-73	PO-72	Standard
	***Group***	TB	TB	TB	LTBI	LTBI	LTBI	Uninfected	Uninfected	Uninfected	Mix
	***TMT-Label***	126	127 N	127 C	128 N	128 C	129 N	129 C	130 N	130 C	131
	***BCG vaccination***	Yes	No	No	No	Yes	No	No	No	No	n.a.
***TMT 4 Serum***	***SAMPLE***	PO-76	PO-90	PO-53	PO-66	PO-101	PO-87	PO-75	PO-77	PO-79	Standard
	***Group***	TB	TB	TB	LTBI	LTBI	LTBI	Uninfected	Uninfected	Uninfected	Mix
	***TMT-Label***	126	127 N	127 C	128 N	128 C	129 N	129 C	130 N	130 C	131
	***BCG vaccination***	No	No	No	No	No	Yes	No	Yes	No	n.a.
***TMT 5 Serum***	***SAMPLE***	PO-102	PO-67	PO-29	PO-80	PO-92	PO-86	PO-82	PO-72	PO-78	Standard
	***Group***	TB	TB	TB	LTBI	LTBI	LTBI	Uninfected	Uninfected	Uninfected	Mix
	***TMT-Label***	126	127 N	127 C	128 N	128 C	129 N	129 C	130 N	130 C	131
	***BCG vaccination***	No	Yes	No	Yes	Yes	No	Yes	No	No	n.a.

### TMT 10plex labeling

Samples were selected for the quantitative proteomics study according to their protein concentration and integrity. Labelling and fractionation of the protein samples were done as previously described^27^. Briefly, 100 µg of each individual sample was resuspended in a final volume of 100 µL of 0.1 M TEAB buffer solution, reduced/alkylated and digested with trypsin for 16 h at 37 °C. Labeling with TMT 10plex reagents (Thermo Fisher Scientific, San Jose, CA, US) was performed following the manufacturer’s indications.

### Peptide fractionation by high-pH reversed phase

Dried aliquots of 100 µg were reconstituted in 300 µL of trifluoroacetic acid (TFA) and the peptides were fractionated using the High-pH Reversed Phase Fractionation Kit (Thermo Fisher Scientific) following the manufacturer’s instructions. The peptide concentration in the resulting eight fractions was determined using the Quantitative Colorimetric Peptide Assay (Thermo Fisher Scientific).

### LC analysis and orbitrap-elite settings

One microgram of each fraction was injected and analyzed by LC-MS/MS using a Proxeon EASY-nLC II liquid chromatography system (Thermo Fisher Scientific) coupled to an LTQ-Orbitrap Elite (Thermo Fisher Scientific). Separation of the peptides was performed on an RP column (EASY-Spray column, 50 cm × 75 μm ID, PepMap C18, 2 μm particles, 100 Å pore size, Thermo Fisher Scientific) with a 10 mm precolumn (Accucore XL C18, Thermo Fisher Scientific) using 0.1% formic acid (mobile phase A) and 98% ACN with 0.1% formic acid (mobile phase B). A 240 min linear gradient from 5 to 35% B was applied at a flow rate of 300 nL per min. Ionization was performed in an NSI source using a spray voltage of 1.95 kV and a capillary temperature of 275 °C. The peptides were analyzed in positive mode (1 μscan; 400–1600 amu), followed by 10 data-dependent HCD (high-energy collision dissociation) MS/MS scans (1 μscans), using a normalized collision energy of 38% at a resolution of 30,000 and an isolation width of 1.5 amu. Dynamic exclusion was enabled with a repeat count of 1, a repeat duration of 30 s, a duration of the exclusion of 80 s, and a relative exclusion width of 10 ppm. Unassigned charged ions were excluded from the analysis.

### Mass spectrometry data processing

Xcalibur 3.1 software (Thermo Fisher Scientific) was used for data acquisition, raw file generation, inspection of the chromatography profile and confirmation of the labeling of the peptides. After that, protein identification and quantification were performed using Proteome Discoverer 2.1 software (Thermo Fisher Scientific). Peak lists were generated with a precursor signal-to-noise ratio of 1.5, and default settings were used to search the latest Human UniProtKB/SwissProt Release (2018_10 with 551,681 entries) with the Sequest algorithm. The enzyme specificity was set to trypsin, and one missed cleavage was tolerated. TMT-labeling and carbamidomethylation of cysteines were set as fixed modifications, whereas oxidation of methionines and N-terminal acetylation were set as variable modifications. The precursor ion mass tolerance was set to 7 ppm, and the product ion mass tolerance was set to 0.06 Da. A decoy database search was performed to determine the peptide false discovery rate (FDR) with the Target Decoy PSM (peptide-spectrum matches) Validator Module. Quantification was performed using a Quantification Module, and normalization was performed against the total peptide amount. A 1% peptide FDR threshold was applied.

Samples were categorized by the patient type (TB, LTBI and uninfected contacts and internal standard). Quantification jobs were alternatively launched using a) the patient type option for the global analysis and b) the individual ratios option for the nonparametric statistical analysis.

### Protein identification and quantification

For the Venn diagram representation, a list of proteins from each TMT experiment was quantified and at least one unique peptide was loaded in the InteractiVenn tool^28^. For ratio dispersion representation, all the ratios for all the proteins quantified in the five TMT experiments were extracted from the Proteome Discoverer 2.1 software using the “export to Excel” option, and dispersion diagrams were performed using XLSTAT software.

### Statistical analysis of the TMT quantification ratios

Only proteins identified and quantified in the five TMT experiments were considered for statistical analysis. Briefly, the protein ratios (45 for each comparison; 9 ratios per TMT experiment, 5 TMT experiments) were imported into R commander console (statistical package included in R 3.5.1 software) and represented in box diagrams. For each protein, data were used to analyze the differences between the three studied ratios (45 ratios uninfected/LTBI, 45 ratios uninfected/TB and 45 ratios TB/LTBI) by applying a Kruskal-Wallis test, since we observed that the quantification ratios did not follow a normal distribution. Differences in the modulation were considered significant when p-value ≤ 0.001.

The final list (UniProt accession number) of significantly modulated proteins was analyzed using String 10.1 software for the determination of pathways and biological processes modulated in each group of participants.

### ELISA of selected proteins

Serum levels of selected proteins were measured in the verification cohort using the following ELISA kits (Abcam): ab99995 (CRP), ab108852 (A1AGP1), ab219048 (HPT), ab108911 (TRFE) and ab202405 (KLK1B). Briefly, serum samples (twenty-one active TB patients and fifteen LTBI contacts) were diluted accordingly in the supplied sample diluent, and immunodetection was performed following the manufacturer’s instructions. Absorbance was recorded in triplicate at 450 nm in a Multiskan GO Plate Reader (Thermo Fisher Scientific). Statistical analysis (Kruskal-Wallis) and diagrams were performed using R commander console and GraphPad software.

### Nephelometry analysis of APOA1 in serum samples

APOA1 serum levels were measured in the verification cohort using an Immage^TM^ nephelometer (Beckman-Coulter) and the corresponding APOA1 reagent kit (#4464110, Beckman Instruments, Inc.) following the manufacturer’s instructions. Statistical analysis and diagrams were performed using R commander console and GraphPad software.

### Receiver operating characteristic (ROC) analysis of the targeted analysis of selected proteins

The diagnostic accuracy of the six selected proteins were assessed by the receiver operator characteristics (ROC) curve analysis. The cut-off values for each parameter were determined by the highest Youden Index in order to maximize the sensitivity and specificity of the test. Statistical analysis was performed using GraphPad Prism version 6.0 for Windows (GraphPad Software; CA, USA) and IBM SPSS version 23 for windows (SPSS Inc., Chicago, Ill., USA).

## Results

### Protein identification and quantification

**Figure 1:**
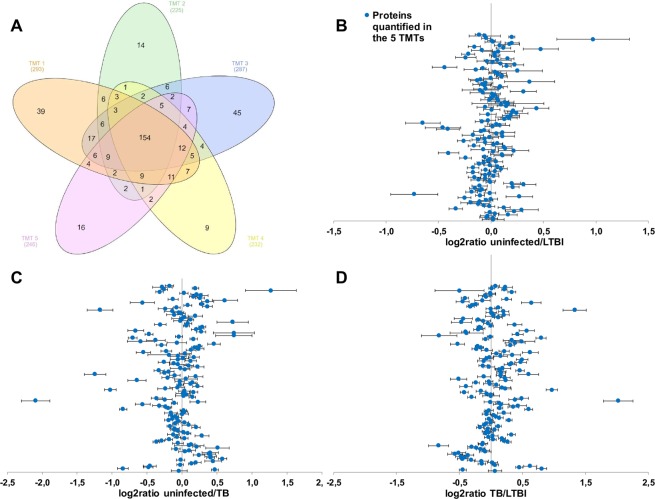
Summary of the quantitative shotgun proteomic study. Venn diagram representation of the five TMT experiments (**A**). A total of 418 proteins were identified and quantified with at least one unique peptide in the whole study. 154 of them were common for the five TMT experiments. Dispersion diagrams for the 154 common proteins (**B**–**D**) representing the mean of the log2 ratio and the standard error of the mean (SEM).

**Figure 2:**
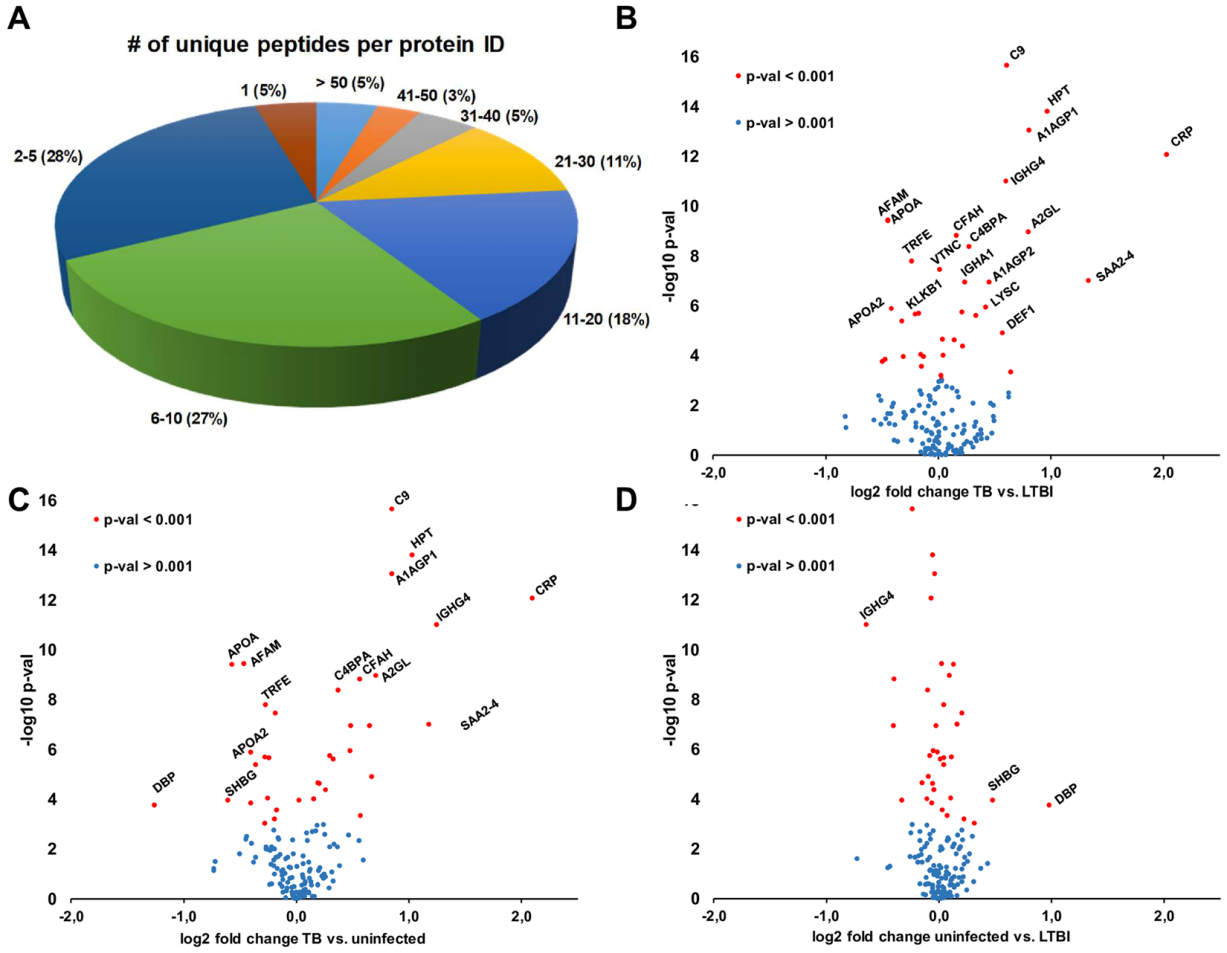
Summary of the number of unique peptides quantified per protein (**A**). Ninety-five percent of the proteins were quantified with at least two unique peptides. Volcano-plot representations of the statistical analysis of the quantification ratios. A specific proteomic signature is detected when comparing active TB patients versus both LTBI (**B**) and uninfected (**C**) contacts. No apparent specific signature is detected when comparing uninfected versus LTBI contacts (**D**). Significance is considered when p-value ≤ 0.001.

Proteomic datasets were deposited in the MassIVE repository (www.massive.ucsd.edu). Raw and processed files (SERUM_DATA_FILES_EMI_TB_PROTEOMICS; #MSV000083645) are public and freely accessible. Figure 1 represents the global identification results. The number of proteins identified in each TMT with at least one unique peptide range from 293 in TMT1 to 225 in TMT2 (Fig. 1A), whereas a total of 154 proteins were identified and quantified in the five TMT experiments with at least one unique peptide, 147 of them with at least two unique peptides (Fig. 2A and Supplementary Information). Dispersion diagrams of the quantification ratios show slight differences between uninfected versus LTBI participants (Fig. 1B) and more evident differences between active TB patients versus both uninfected and LTBI contacts (Fig. 1C,D).

### Specific serum proteomic signature of active TB patients, LTBI contacts and uninfected contacts

A nonparametric Kruskal-Wallis statistical analysis was applied to the quantification ratios for all the proteins. Volcano-plot representations are shown in Fig. 2. Differences were detected between active TB patients and both uninfected and LTBI contacts (Fig. 2A,B), whereas no detectable differences were detected when comparing LTBI versus uninfected contacts.

Examples of proteins that accumulated in the serum of active TB patients include C-reactive protein (CRP), haptoglobin (HPT) alpha-1-acid glycoprotein 1 (A1AGP1), complement component C9 (C9), neutrophil defensin 1 (DEF1) and serum amyloid P component (SAA2–4). Volcano-plot representation of the quantification ratios showed no apparent differences in the serum protein content when comparing LTBI versus uninfected contacts.

In contrast, proteins such as apolipoprotein A (APOA1 and 2), serotransferrin (TRFE) and plasma kallikrein (KLK1B) were decreased in the serum of active TB patients (Fig. 2A,B). Similarly, the proteins that were diminished in the serum of active TB patients presented no differences when comparing LTBI versus uninfected contacts (Fig. 2).

### Pathway and interaction analysis of modulated proteins

**Figure 3:**
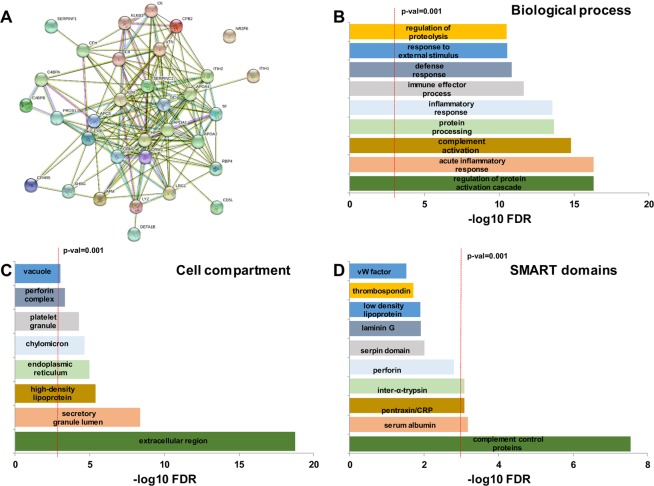
Interaction and pathway analysis of the proteins detected as modulated in active TB patients. String 10.1 analysis show a strong interaction network (**A**) between those proteins. Statistical pathway analysis (**B**–**D**) show that most of the proteins play roles in defense against pathogens, complement activation and inflammation.

**Figure 4:**
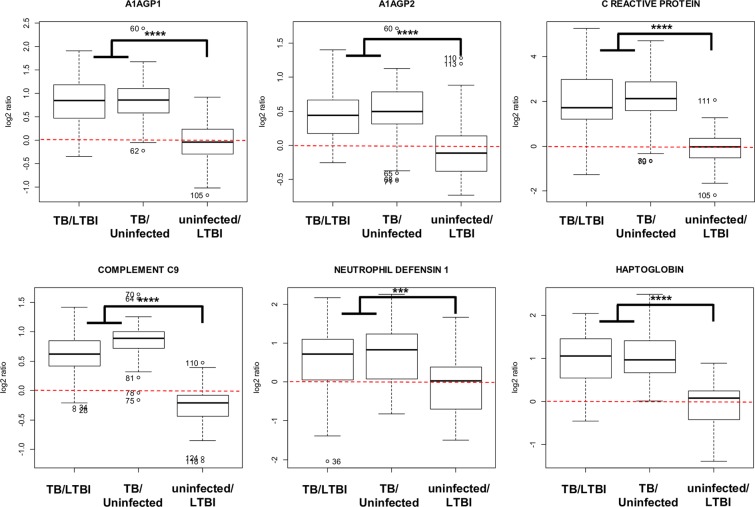
Box-plot representation of selected proteins that are detected as over-represented in the serum of active TB patients versus both LTBI and uninfected contacts but are not modulated when comparing uninfected versus LTBI contacts. ****p-value ≤ 0.00001; ***p-value ≤ 0.001.

**Figure 5:**
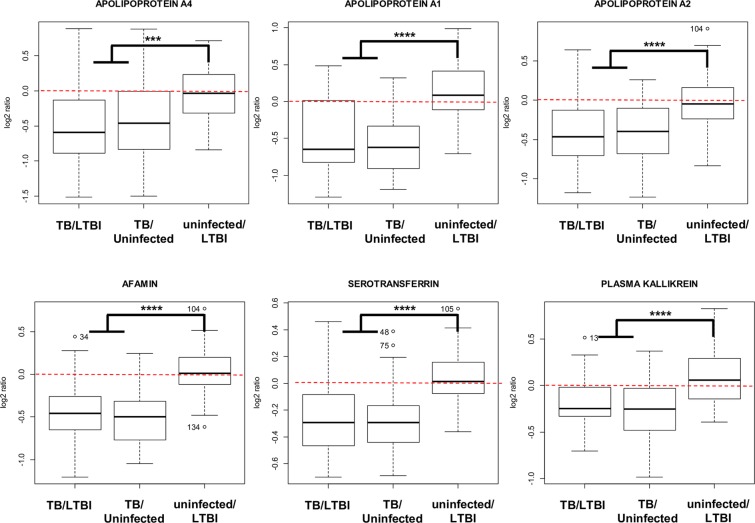
Box-plot representation of selected proteins that are detected as decreased in the serum of active TB patients versus both LTBI and uninfected contacts but are not modulated when comparing uninfected versus LTBI contacts. ****p-value ≤ 0.00001; ***p-value ≤ 0.001.

String 10.1 analysis showed a strong interaction network between the set of modulated proteins (Fig. 3A). Enrichment analyses by biological processes, cell compartment and SMART domains were performed (Fig. 3B–D), showing that most of the proteins modulated in active TB patients are secreted and play roles in complement activation, acute-phase response and modulation of inflammation. Box plot diagrams of the quantification ratios show that this set of proteins is specifically modulated in the serum of active TB patients, while no differences are detected when comparing LTBI and uninfected contacts (Figs. 4 and 5).

### Targeted measurement of selected candidates in serum samples

**Figure 6:**
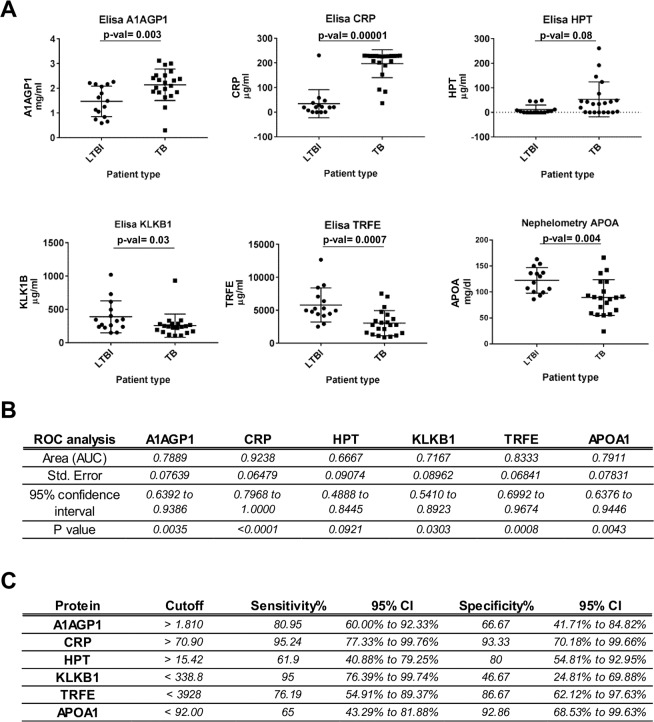
Targeted measurement of the serum levels of selected candidates (three over-represented and three decreased in active TB patients) in an independent cohort (verification cohort, Maputo region, Mozambique). Significant (p-val≤ 0.05) modulation is detected for five out of the six proteins (**A**). ROC (**B**) and Youden index (**C**) analysis was done for the six targets to investigate their discriminatory potential between active TB patients and latently infected contacts.

ELISA and nephelometry techniques were used for verification in a second cohort due to the clinical relevance of identified proteins. A1AGP1, CRP and HPT were selected as proteins overrepresented in active TB patients versus both LTBI and uninfected contacts, whereas KLKB1, TRFE and APOA were selected as proteins diminished in active TB patients versus both LTBI and uninfected contacts. Statistical analysis showed similar results to those obtained for the six proteins in the shotgun proteomics phase (Fig. 6A). Significant differences (p-value ≤0.01) were observed for A1AGP1, CRP, TRFE and APOA. Less significant differences (p-value ≤0.05) were obtained for KLKB1, and no significant differences (p-value = 0.08) were observed for HPT.

Receiver Operating Characteristic (ROC) analysis showed that five out of the six proteins present AUC (area under the curve) values higher than 0.7 (Fig. 6B). Two of them, CRP (AUC= 0.92) and TRFE (AUC= 0.83), presented the best balance between sensitivity and specificity, as revealed by Youden index analysis (Fig. 6C).

## Discussion

The host innate and acquired immune processes activated during MTB infection and disease progression are still not fully understood. Biomarker discovery in biological fluids using high-resolution proteomics allows researchers to identify the modulation of cellular pathways when comparing different disease progression states. In the previous work, we focused on the analysis of nasal/oral fluid samples to obtain a deeper understanding of the innate immune processes implicated in resistance to the initial MTB infection^27^. In the present study, by analyzing serum samples of active TB patients and their contacts, our aim was to focus on the mechanisms activated systematically in the host during the progression of the disease.

A total of 418 serum proteins were quantified in our study. At first, it might seem that the number of total quantified proteins using our approach was not high, but this result is typical of non-depleted serum samples. Depletion of abundant proteins using commercially available antibody-coated LC columns or cartridges^29^ as well as other approaches, such as those based on the use of combinatorial hexapeptide ligand libraries^30^, enables a higher coverage of the serum and plasma proteome in mass spectrometry-based studies but at the cost of losing information from highly abundant proteins and protein complexes. In a previous multilaboratory experiment (Proteored Multicenter Experiment 6), several groups were able to identify more than one hundred different proteins in the “bound” fraction of a immune-depleted serum sample with a commercial column supposed to bind only the 20 most abundant serum proteins^31^. In the present study, we took the view that information coming from those abundant proteins is important and should be also analysed. In future studies, we also think that a larger coverage of the serum proteome could be obtained by increasing the level of peptide fractionation, using off-gel-based techniques^32^. However, the number of proteins quantified in the present study are in concordance with very recent serum-based studies. Using MALDI-TOF mass spectrometry (no tryptic digestion and no fragmentation) Zhang *et al*.^33^ found 236 serum “protein peaks” differentially expressed between patients with Intestinal Tuberculosis (ITB) and Crohn´s Disease (CD). In another study by Arya *et al*.^34^, 132 and 68 serum extracellular vesicle (EVs) proteins from active TB patients were identified when compare to non-tuberculosis and healthy patients. The number of identified proteins were higher (more than seven hundred with at least one unique peptide) in the study by Chen *et al*.^35^ by analyzing immune-depleted serum samples. Finally, Liu *et al*.^36^ were able to identify one thousand proteins in plasma samples using label-free quantification of severe and mild TB patients versus healthy individuals with no exposure to MTB.

Regarding protein identification and quantification, we obtained good reproducibility when comparing the data from five different TMT experiments. A total of 154 proteins are common to the five biological replicates and 147 of them were quantified with at least two unique peptides (Fig. 2A and supplementary info). The standard error of the mean (SEM) of the ratios is low for most of them (Fig. 1B–D).

Kruskal-Wallis test was applied to detect significantly modulated proteins when comparing different progression states of the disease (Fig. 2B–D). Uninfected contacts remained free of MTB infection despite high degree exposure to an active TB patient (Table 1). We have recently identified some components of the innate immune response that specifically accumulate in the sputum of these uninfected individuals when compared with LTBI contacts^27^, indicating the importance of nasal/oral secretions as a first barrier to fighting the initial entry of MTB. In the present study, using the same approach, we were unable to detect any serum protein significantly accumulated in uninfected contacts versus LTBI contacts (Fig. 2D). In contrast, a set of proteins were significantly accumulated in active TB patients versus both LTBI and uninfected contacts (Fig. 2B,C).

Interaction network and pathway analysis showed that the specific protein signature of active TB patients was characterized by an accumulation of proteins related to complement activation, inflammation and modulation of the immune response (Fig. 3). Individual box plot diagrams representing the three kind of ratios show the specificity for modulated proteins (Figs. 4 and 5). For instance, proteins, such as C-reactive protein, haptoglobin and alpha-1 acid glycoprotein- 1 and −2 (ORM-1 and -2), were specifically accumulated in active TB patients, whereas no differences were detected when comparing LTBI and uninfected contacts (Fig. 4). All these proteins were previously identified as accumulated in serum samples of TB patients with antibody-based techniques^37,38^, by label-free mass spectrometry^36^, and more recently in saliva and sputum samples^27^. To the best of our knowledge, other proteins, such as neutrophil defensin 1 and lysozyme C, have not been previously described as being accumulated in the serum of active TB patients.

**Figure 7:**
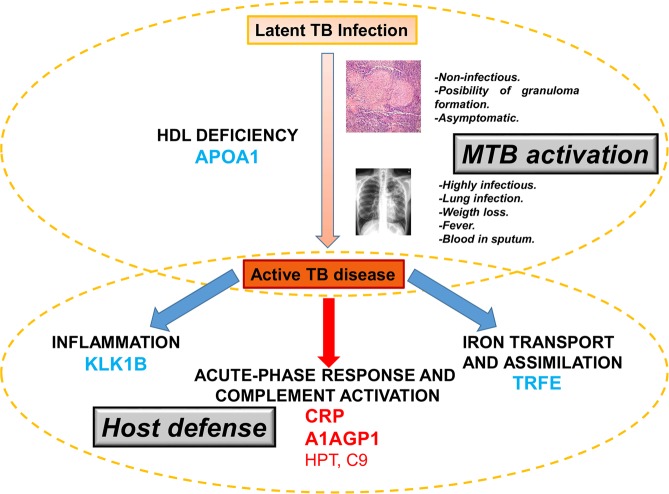
Schematic representation of the processes and proteins detected as modulated during the progression of the TB from a latent asymptomatic infection to an active disease. Proteins in blue are decreased in active TB patients whereas proteins in red are increased in this group versus both LTBI and uninfected contacts. Proteins in bold were also detected as significantly modulated in an independent cohort by targeted antibody-based techniques.

Furthermore, a decrease in a small subset of proteins, including apolipoproteins A1, A2 and A4, serotransferrin and plasma kallikrein, was revealed as characteristic of active TB patients (Fig. 5). The ApoA complex is a central component of high density lipoproteins (HDL) and is one of the main components responsible for lipid transport from tissues to the liver^39^. A decrease in ApoA1 was associated with HDL deficiency type 2 or hypercholesterolemia more than two decades ago^40^; interestingly, a decrease in ApoA1 has been more recently reported to cause an impairment of the immune response against TB^41^. In relation to this finding, ApoA was detected to be increased during rifampicin treatment of active TB in humans^42^ and formed a complex with isoniazid during the treatment in a TB mouse model^43^. At this point, it is important to remark that the serum samples used in the present study were collected before patients underwent anti-TB treatment, so the serum levels of ApoA1 are not influenced by the uptake of anti-TB or any other drugs. This finding is in concordance with a previous quantitative proteomics study in which ApoA was found to be increased after anti-TB drug treatment in cured *versus* untreated tuberculosis patients^44^, thus strongly indicating that rifampicin/isoniazid treatment increases the serum levels of this protein. A possibility is that LTBI contacts with decreased levels of ApoA1 could be more susceptible to developing active TB than those presenting normal levels of this protein (Fig. 7). Further investigations are needed to explore the putative implications of this result in the treatment of the disease.

In addition, iron assimilation of MTB from the host has been revealed as a key pathway for the progression of the disease^45^. Specifically, MTB acquisition of iron from serotransferrin has been reported to be decreased by IFN-gamma treatment^46^. Patients with hemochromatosis, also known as iron overload, have been previously associated with more severe TB disease^47^. The decrease in serotransferrin levels as a host mechanism of defense against TB progression *via* iron deprivation represents an attractive possibility to be investigated in the future.

Plasma kallikreins are a group of serine proteases involved in inflammation and autoimmune response processes^48,49^. KLK1 was previously described by genome-wide transcriptome profiling as downregulated in MTB-stimulated peripheral blood mononuclear cells (PBMCs) from patients with TB; thus, KLK1 was previously used in combination with other markers to discriminate active TB patients from LTBI individuals and healthy controls^50^.

Verification of selected proteins in a second cohort was performed using antibody-based targeted techniques since these approaches are still considered the gold standard for clinical validation despite the promising development of multiplexing immunoassays^51^. The targeted measurement of the selected proteins was in agreement with the shotgun proteomic data, and five out of the six proteins were detected as significantly modulated in active TB patients versus LTBI contacts (Fig. 6A). ROC analysis showed that the serum levels of those five proteins could have a potential for discriminating between active TB and latent infected contacts. In particular, CRP and TRFE display a good balance between sensitivity and specificity (Fig. 6B,C and Supplementary Data 2). However, the putative discriminatory potential of those targets is still based on preliminary data and further investigation using bigger cohorts including other non-TB respiratory diseases are needed.

TB burden is especially dramatic in sub-Saharan countries^52^; Mozambique, as the country of origin in the second cohort is from this region and, therefore, verification of the initial quantitative proteomics study in this second group of participants from a distant geographical region increases the strength of our findings and suggests a common host-acquired immune response to TB. Recently, mass spectrometry has been used to study the effect of phosphorylation in mycobacterial proteins known to be involved in MTB virulence^53^. Although, MTB strain identification was not performed during patient diagnosis, it would be of interest to investigate whether or not different MTB strains possessing distinct pathogenicity traits promote similar defense mechanisms in the host.

We think that the findings presented in our study could have important implications for treating MTB and contribute to our understanding of the host defense mechanisms against TB. To summarize, lipid transport, iron assimilation acute-phase response and inflammation have all been identified as modulated pathways when comparing latent TB infection versus active TB disease (Fig. 7). We believe that these findings carry significant biological relevance and will help to better understand MTB pathogenesis and the host innate response, potentially aiding the rational vaccine design and testing for future studies.

## Supplementary information

Dataset 1.

Dataset 2.

## Data Availability

Raw and processed files (SERUM_DATA_FILES_EMI_TB_PROTEOMICS; #MSV000083645) and statistical analysis are publicly and freely accessible from the MassIVE Repository (www.massive.ucsd.edu).
